# Supplementary material for: Linoleic acid improves PIEZO2 dysfunction in a mouse model of Angelman Syndrome
Source: Nat Commun. 2023 Mar 1;14:1167. doi: 10.1038/s41467-023-36818-0 (PMC9977963; doi:10.1038/s41467-023-36818-0)
Supplement: Supplementary file 1 — Supplementary information [file 41467_2023_36818_MOESM1_ESM.pdf]

SUPPLEMENTARY INFORMATION

Linoleic Acid Improves PIEZO2 Dysfunction in a mouse model of Angelman Syndrome

Romero, Caires et al.

SUPPLEMENTARY FIGURES

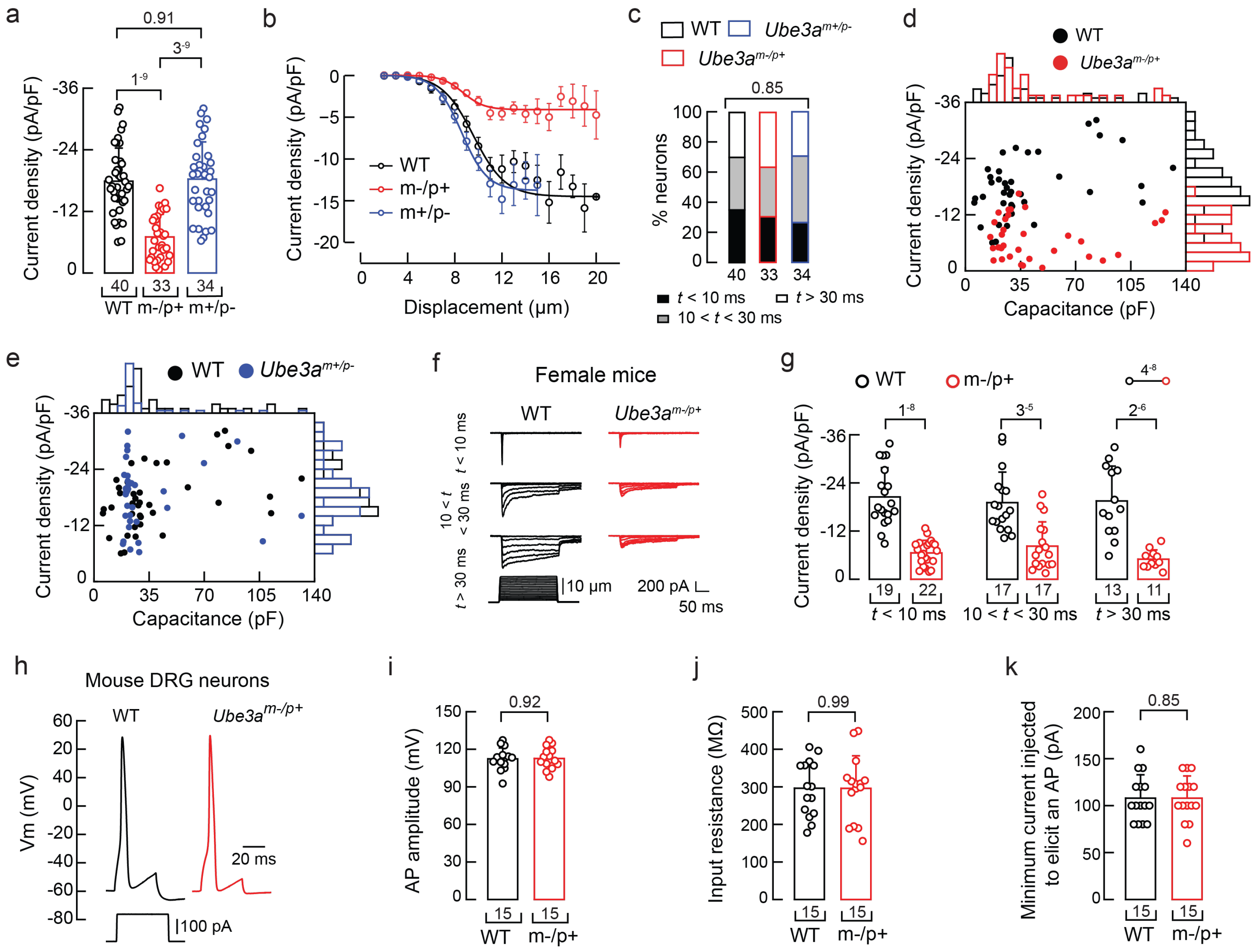

Supplementary Fig. 1. Mechanocurrents and inactivation distribution of DRG neurons from AS mice. **a** Current densities elicited by maximum displacement of WT, *Ube3a*$^{m–/p+}$, and *Ube3a*$^{m+/p–}$ mouse DRG neurons. Bars are mean ± SD. Kruskal-Wallis (H = 47.17; p = 5.73^{-11}) and Dunn's multiple comparisons test. **b** Current density responses to various displacements of WT, *Ube3a*$^{m–/p+}$, and *Ube3a*$^{m+/p–}$ mouse DRG neurons. A Boltzmann function was fitted to the data. Each circle represents the mean ± SEM. For WT, n = 40, 40, 40, 40, 40, 40, 39, 39, 38, 35, 25, 18, 14, 10, 7, 4, 4, 3, 2, per displacement. For *Ube3a*$^{m–/p+}$, n = 33, 33, 33, 33, 33, 33, 33, 33, 33, 31, 26, 18, 14, 8, 8, 4, 3, 3, 3, per displacement. For *Ube3a*$^{m+/p–}$, n = 34, 34, 34, 34, 34, 34, 34, 31, 28, 21, 9, 6, 4, per displacement. **c** Proportions of neurons classified by their time constant of inactivation of mechano-activated currents elicited by maximum displacement of WT, *Ube3a*$^{m–/p+}$, and *Ube3a*$^{m+/p–}$ mouse DRG neurons. Chi-square test (χ^2 = 1.37; p = 0.85). **d** Scatter plot of current density *vs.* capacitance of WT and *Ube3a*$^{m–/p+}$ mouse DRG neurons. Top and right display the corresponding marginal histograms. **e** Scatter plot of current density vs. capacitance of WT and *Ube3a*$^{m+/p–}$ mouse DRG neurons. Top and right display the corresponding marginal histograms. **f** Representative whole-cell patch-clamp recordings elicited by mechanical stimulation (at -60 mV) of rapidly, intermediate, and slowly inactivating currents of WT and *Ube3a*$^{m–/p+}$ DRG neurons from female mice. **g** Current densities elicited by maximum displacement of DRG neurons from female mice, classified by their time constant of inactivation. Bars are mean ± SD. Two-way ANOVA (F = 103.04; p = 1.11^{-16}) and Tukey multiple-comparisons test. **h** Representative current-clamp recording of membrane potential changes elicited by current injection in WT and *Ube3a*$^{m–/p+}$ mouse DRG neurons. **i** Action potential amplitudes evoked by current injection in WT and *Ube3a*$^{m–/p+}$ mouse DRG neurons. Bars are mean ± SD. Two-

tailed unpaired t-test ($t = 0.10$). **j** Input resistance of WT and *Ube3a*$^{m-/p+}$ mouse DRG neurons. Bars are mean ± SD. Two-tailed unpaired t-test ($t = 0.013$). **k** Minimum current injected that elicited action potentials in WT and *Ube3a*$^{m-/p+}$ mouse DRG neurons. Bars are mean ± SD. Two-tailed Mann-Whitney test (U = 107.5). n is denoted in each panel. Post hoc *p*-values are denoted above the bars. Source data are provided as a Source Data file.

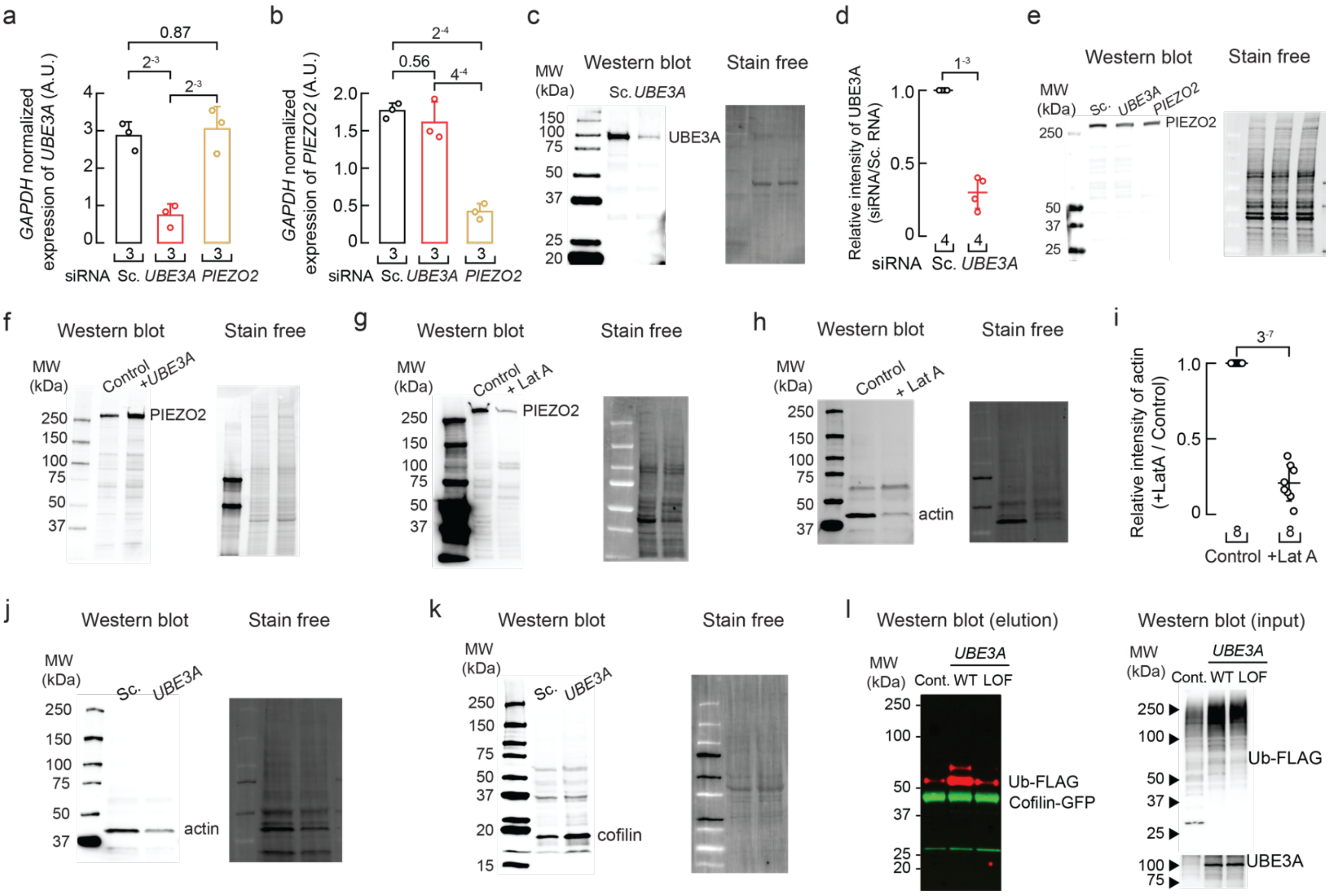

Supplementary Fig. 2. RT-qPCR and western blots after *UBE3A* knockdown or overexpression, latrunculin A treatment, and cofilin ubiquitination.

a Relative normalized expression of *UBE3A* for MCC13 cells transfected with scrambled (Sc.), *UBE3A*, or *PIEZO2* siRNAs. Bars are mean ± SD. One-way ANOVA (F = 25.37; p = 0.0012) and Tukey multiple-comparisons tests. **b** Relative normalized expression of *PIEZO2* for MCC13 cells transfected with scrambled (Sc.), *UBE3A*, or *PIEZO2* siRNAs. Bars are mean ± SD. One-way ANOVA (F = 50.94; $p = 1.72^{-4}$) and Tukey multiple-comparisons tests. **c** Representative western (anti-UBE3A) and stain-free blots of the cytosolic fractions of MCC13 cells transfected with scrambled or *UBE3A* siRNAs (48 h). **d** Mean/scatter-dot plot showing relative intensities of UBE3A protein normalized to the level of UBE3A in the Sc. group. Lines are mean ± SD. Two-tailed one sample *t*-test (t = -12.7). **e** Representative western (anti-PIEZO2) and stain-free blots of the membrane fractions of MCC13 cells transfected with scrambled, *UBE3A*, or *PIEZO2* siRNAs (48 h), from six independent cultures. **f** Representative western (anti-PIEZO2) and stain-free blots of the membrane fractions of MCC13 cells transfected with control or *UBE3A* plasmids, from seven independent cultures. **g** Representative western (anti-PIEZO2) and stain-free blots of the membrane fractions of MCC13 cells treated with latrunculin A (1 µM; 24 h), from eight independent cultures. **h** Representative western (anti-actin) and stain-free blots of the cytoskeletal fractions of MCC13 cells treated with latrunculin A (1 µM; 24 h). **i** Mean/scatter-dot plot

showing relative intensities of actin protein normalized to the level of actin in the control group. Lines are mean ± SD. n is denoted below the circles. One sample *t*-test (t = -18.73). **j** Representative western (anti-actin) and stain-free blots of the cytoskeletal fractions of MCC13 cells transfected with scrambled or *UBE3A* siRNAs (48 h), from eight independent cultures. **k** Representative western (anti-cofilin) and stain-free blots of the cytosolic fractions of MCC13 cells transfected with scrambled or *UBE3A* siRNAs (48 h), from five independent cultures. **l** Left, representative western blot of pulldown GFP-tagged Cofilin from HEK293T cells transfected with a control vector (Ctrl), wild-type *UBE3A* (WT), or a catalytically inactive *UBE3A* (LOF), from four independent preparations. The ubiquitinated (Ub) fraction (red) was monitored with an anti-FLAG antibody. Right, representative western blots (anti-FLAG and anti-UBE3A, respectively) of the input fractions (lysate), from four independent preparations. n is denoted in each panel. Post-hoc *p*-values are denoted in the corresponding panels. Source data are provided as a Source Data file.

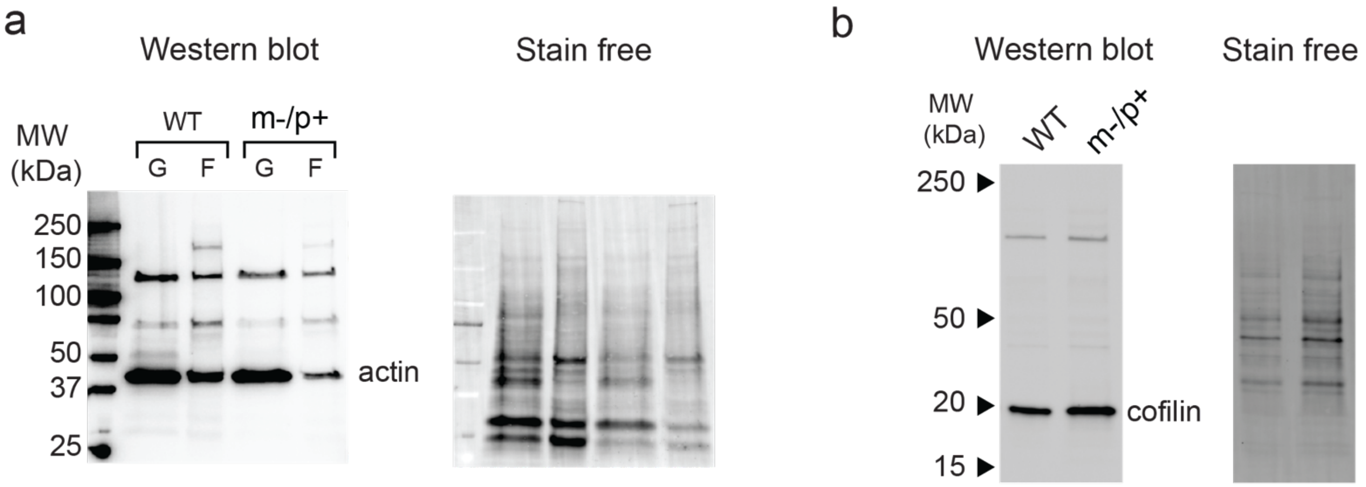

Supplementary Fig. 3. G-actin, F-actin, and cofilin content of *Ube3a*$^{m–/p+}$ DRGs.

a Representative western (anti-actin) and stain-free blots of soluble and insoluble actin (G and F, respectively) of WT and *Ube3a*$^{m–/p+}$ DRGs, from 12 mice. **b** Representative western blot (anti-cofilin) and stain-free blots of the cytosolic fractions of WT and *Ube3a*$^{m–/p+}$ DRGs, from 8 mice.

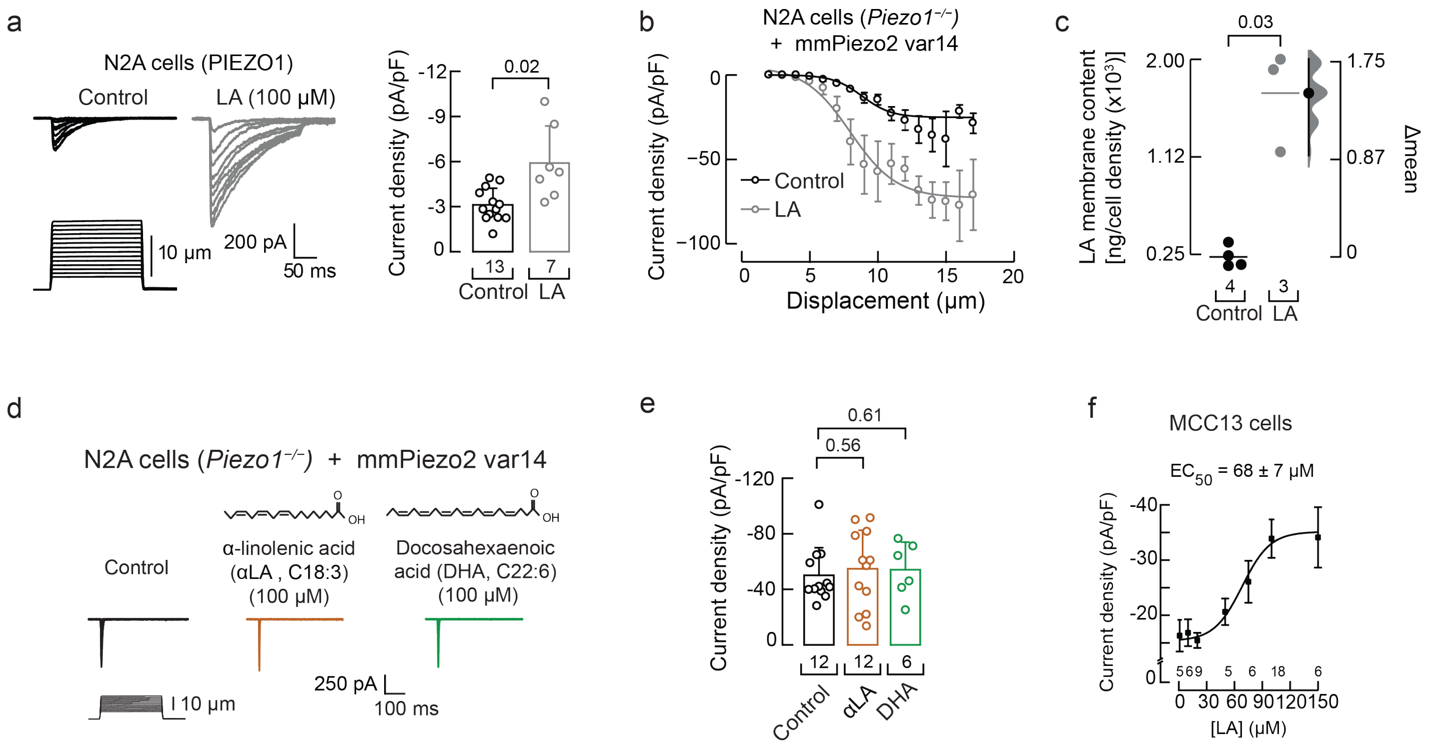

Supplementary Fig. 4. Effect of PUFAs on PIEZOs channel function in N2A cells.

a Left, representative whole-cell patch-clamp recordings of currents elicited by mechanical stimulation (at -60 mV) in control or LA (100 µM; 24 h)-treated N2A cells (endogenously expressing *Piezo1*). Right, current densities elicited by maximum displacement of control or LA-treated N2A cells (endogenously expressing *Piezo1*). Bars are mean ± SD. Two-tailed unpaired *t*-test with Welch's correction ($t = 2.84$). **b** Current density responses to various displacements of control vs. LA-treated *Piezo1*$^{-/-}$ N2A cells transfected with *Piezo2* variant 14 (var14). A Boltzmann function was fitted to the data. Each circle represents the mean ± SEM. For control, n = 12, 12, 12, 12, 12, 12, 12, 12, 11, 11, 9, 8, 7, 5, 3, 3, per displacement. For LA-treated cells, n = 17, 17, 17, 17, 17, 17, 17, 17, 16, 14, 13, 12, 11, 10, 6, 5, per displacement. **c** Gardner–Altman estimation plot showing the mean difference in LA membrane content in control and LA (100 µM)-treated N2A cells, as determined by LC-MS. Raw data are plotted on the left axis. The mean difference, on the right, is depicted as a dot; the 95% confidence interval is indicated by the ends of the vertical error bars. Two-tailed unpaired *t*-test with Welch's correction ($t = 5.47$). **d** Representative whole-cell patch-clamp recordings of control, αLA, and DHA (100 µM; 24 h)-treated *Piezo1*$^{-/-}$ N2A cells transfected with *Piezo2* var14. **e** Current densities elicited by maximum displacement of control, αLA, and DHA (100 µM; 24 h)-treated *Piezo1*$^{-/-}$ N2A cells transfected with *Piezo2* var14. Bars are mean ± SD. Kruskal-Wallis ($H = 0.43$; $p = 0.81$) and Dunn's multiple comparisons test. **f** Current densities elicited by maximum displacement of MCC13 cells treated with various LA concentrations. A Boltzmann function was fitted to the data. Squares are mean ± SEM. n is denoted in each panel. Post-hoc *p*-values are denoted in the corresponding panels. Source data are provided as a Source Data file.

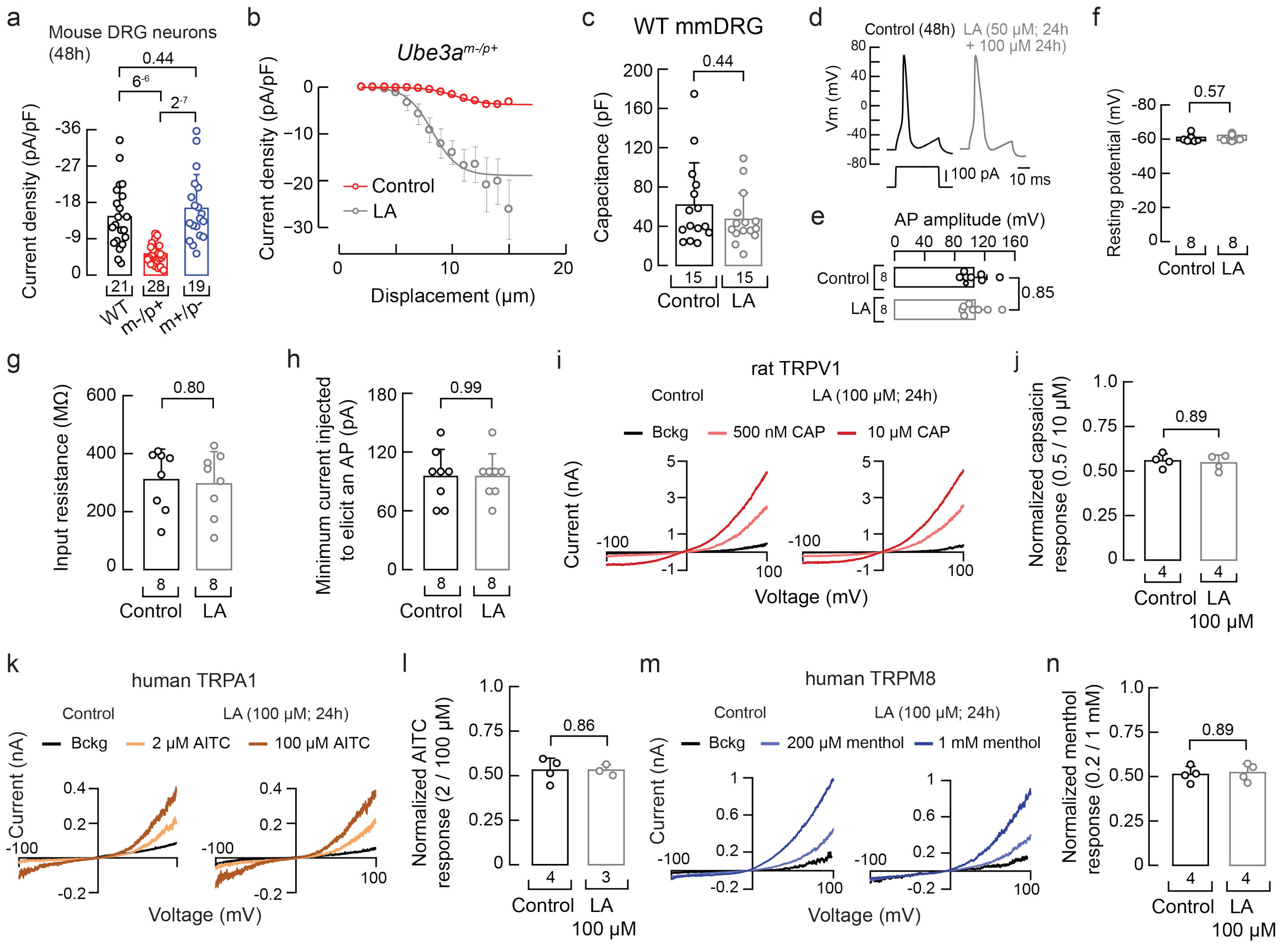

Supplementary Fig. 5. Electrophysiological characterization of neuronal electrical excitability and sensory ion channels in LA-treated cells.

a Current densities elicited by maximum displacement of WT, *Ube3a*$^{m–/p+}$, and *Ube3a*$^{m+/p–}$ DRG neurons recorded after culturing for 48 h. Bars are mean ± SD. Kruskal-Wallis (H = 34.18; p = 3.78^{-8}) and Dunn's multiple comparisons test. **b** Current density responses to various displacements of *Ube3a*$^{m–/p+–}$ mouse DRG neurons supplemented with or without LA. A Boltzmann function was fitted to the data. Each circle represents the mean ± SEM. For control, n = 28, 28, 28, 28, 28, 28, 28, 28, 28, 27, 25, 23, 16, 9, 7, 6, per displacement. For LA-treated cells, n = 28, 28, 28, 28, 28, 28, 28, 27, 26, 23, 17, 13, 10, 8, 5, 5, per displacement. **c** Membrane capacitance of control and LA-treated WT mouse DRG neurons. Bars are mean ± SD. Two-tailed Mann-Whitney test (U = 93). **d** Representative current-clamp recording of membrane potential changes elicited by current injection in control and LA (50 µM for 24 h and 100 µM for another 24 h)-treated WT mouse DRG neurons. **e** Action potential amplitudes evoked by current injection in control and LA-treated WT mouse DRG neurons. Bars are mean ± SD. Two-tailed unpaired *t*-test (*t* = 0.19). **f** Boxplot of the resting potential values recorded immediately after establishing the whole-cell configuration in control and LA-treated WT mouse DRG neurons. Boxplots show mean (square), median (bisecting line), bounds of box (75th to 25th percentiles), outlier range with 1.5 coefficient (whiskers), and minimum and maximum data points. Two-tailed Mann-Whitney test (U = 26). **g** Input resistance of control and LA-treated WT mouse DRG neurons. Bars are mean ± SD. Two-tailed unpaired t-test (*t* = 0.26). **h** Minimum current injected that elicited action potentials in control and LA-treated WT mouse DRG neurons. Bars are mean ± SD. Two-tailed Mann-Whitney test (U = 31.5). **i**

Representative current-voltage relationships of control and LA (100 µM)-treated *Piezo1*$^{-/-}$ N2A cells transfected with rTRPV1 challenged with capsaicin (CAP; 500 nM and 10 µM). Bckg indicates background currents. **j** Normalized capsaicin response (500 nM and 10 µM) of control and LA (100 µM)-treated *Piezo1*$^{-/-}$ N2A cells transfected with rTRPV1. Bars are mean ± SD. Two-tailed Mann-Whitney test (U = 7). **k** Representative current-voltage relationships of control and LA (100 µM)-treated *Piezo1*$^{-/-}$ N2A cells transfected with hTRPA1 challenged with allyl isothiocyanate (AITC; 2 µM and 100 µM). Bckg indicates background currents. **l** Normalized AITC response (2 µM and100 µM) of control and LA (100 µM)-treated *Piezo1*$^{-/-}$ N2A cells transfected with hTRPA1. Bars are mean ± SD. Two-tailed Mann-Whitney test (U = 5). **m** Representative current-voltage relationships of control and LA (100 µM)-treated *Piezo1*$^{-/-}$ N2A cells transfected with hTRPM8 challenged with menthol (200 µM and 1 mM). Bckg indicates background currents. **n** Normalized menthol response (200 µM and 1 mM) of control and LA (100 µM)-treated *Piezo1*$^{-/-}$ N2A cells transfected with hTRPM8. Bars are mean ± SD. Two-tailed Mann-Whitney test (U = 7). n is denoted in each panel. Post hoc *p*-values are denoted above the boxes and bars. Source data are provided as a Source Data file.

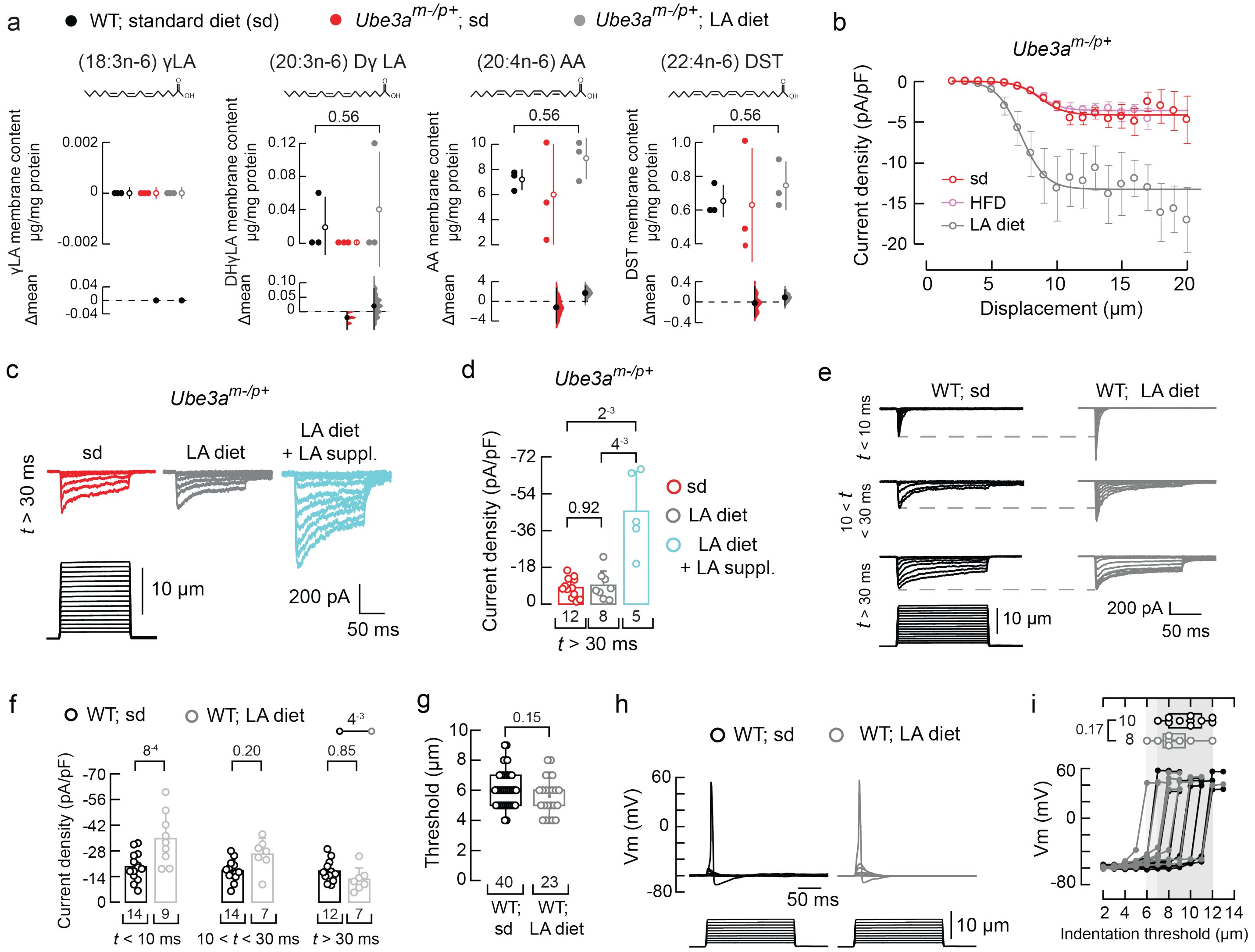

Supplementary Fig. 6. Content of PUFAs downstream of LA in mice fed with a standard or LA-enriched diet.

a Cumming estimation plots showing the mean differences in γ-linolenic acid (γLA), dihomo- γ-linolenic acid (DγLA), arachidonic acid (AA), and docosatetraenoic acid (DST) membrane content in the DRGs of WT and *Ube3a*$^{m–/p+}$ mice fed with a standard diet, as well as *Ube3a*$^{m–/p+}$ mice fed with a LA-enriched diet. Raw data are plotted on the upper axes (n = 3); each mean difference is plotted on the lower axes as a bootstrap sampling distribution. The mean differences are depicted as dots; 95% confidence intervals are indicated by the ends of the vertical error bars. Kruskal-Wallis (for DγLA: H = 1.17 and p = 0.56; for AA: H = 1.16 and p = 0.56; for DST: H = 1.17 and p = 0.56) and Dunn's multiple comparisons tests. n = 3. **b** Current density responses to various displacements of DRG neurons from of *Ube3a*$^{m–/p+}$ mice fed with standard (sd), high-fat (HFD) or LA-enriched (LA diet) diets. A Boltzmann function was fitted to the data. Each circle represents the mean ± SEM. For sd, n = 33, 33, 33, 33, 33, 33, 33, 33, 33, 31, 26, 18, 14, 8, 8, 4, 3, 3, 3, per displacement. For HFD, n = 30, 30, 30, 30, 30, 30, 30, 30, 30, 27, 24, 16, 12, 8, 6, 4, per displacement. For LA diet, n = 38, 38, 38, 38, 38, 38, 38, 36, 33, 26, 24, 20, 19, 16, 13, 11, 10, 9, 7, per displacement. **c** Representative whole-cell patch-clamp recordings elicited by mechanical stimulation (at -60 mV) of slowly inactivating currents in DRG neurons from *Ube3a*$^{m–/p+}$ mice fed with a standard diet, LA-enriched diet, or LA-enriched diet plus LA supplementation using the two-day LA supplementation protocol (50 μM for 24 h and 100 μM for another 24 h). **d** Slowly inactivating current densities elicited by maximum displacement of DRG neurons of slowly inactivating currents in DRG neurons from *Ube3a*$^{m–/p+}$ mice fed with a standard diet, LA-enriched diet, or LA-enriched diet plus LA supplementation using the two-day LA supplementation protocol (50 μM for 24 h and 100 μM for another 24 h). Bars are mean ± SD. Kruskal-Wallis (H = 11.09; p = 3.9^{-4}) and Dunn's multiple comparisons tests. **e** Representative whole-cell patch-clamp recordings elicited by mechanical stimulation (at -60 mV) of rapidly, intermediate, and slowly inactivating currents in DRG neurons from WT mice fed with a standard (sd) or LA-enriched diet. **f** Current densities elicited by maximum displacement of DRG neurons, classified by their time constant of inactivation, from WT mice fed with a standard (sd) or LA-enriched diet. Bars are mean ± SD. Two-way ANOVA (F = 9.1; p = 0.0038) and Tukey multiple-comparisons test. **g** Boxplots show the displacement thresholds required to elicit mechanocurrents in DRG neurons from WT mice fed with a standard (sd) or LA-enriched diet. Boxplots show mean (square), median (bisecting line), bounds of box (75th to 25th percentiles), outlier range with 1.5 coefficient (whiskers), and minimum and maximum data points. Two-tailed Mann-Whitney test (U = 362.5). **h** Representative current-clamp recordings of membrane potential changes elicited by mechanical stimulation of DRG neurons from WT mice fed with a standard (sd) or LA-enriched diet. **i** Membrane potential peak *vs*. mechanical indentation of independent DRG neurons from WT mice fed with a standard (sd) or LA-enriched diet. At the top, boxplots show the displacement threshold required to elicit an action potential in these neurons. Boxplots show mean (square), median (bisecting line), bounds of box (75th to 25th percentiles), outlier range with 1.5 coefficient (whiskers), and minimum and maximum data points. Two-tailed unpaired t-test (t = 1.43). n is denoted in each panel. Post-hoc p-values are denoted in the corresponding panels. Source data are provided as a Source Data file.

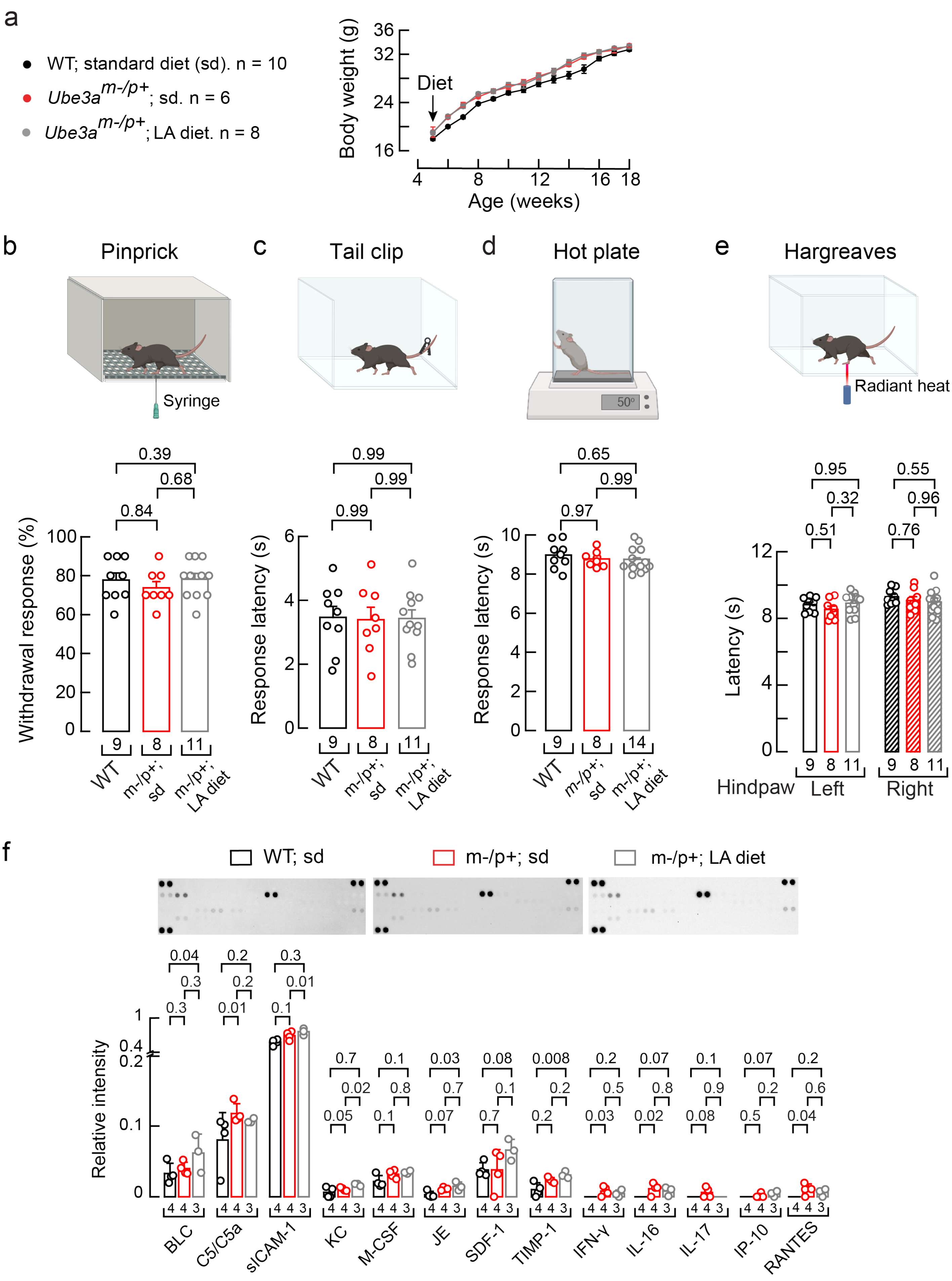

a
WT; standard diet (sd). n = 10
Ube3a m-/p+; sd. n = 6
Ube3a m-/p+; LA diet. n = 8
Body weight (g)
Diet
Age (weeks)
b
Pinprick
Syringe
Withdrawal response (%)
c
Tail clip
Response latency (s)
d
Hot plate
50°
Response latency (s)
e
Hargreaves
Radiant heat
Latency (s)
Hindpaw
Left
Right
WT
m-/p+; sd
m-/p+; LA diet
f
WT; sd
m-/p+; sd
m-/p+; LA diet
Relative intensity
BLC
C5/C5a
sICAM-1
KC
M-CSF
JE
SDF-1
TIMP-1
IFN-γ
IL-16
IL-17
IP-10
RANTES

Supplementary Fig. 7. Body weight, nociceptive responses, and cytokine profiles of mice fed with a standard or LA-enriched diet.

a Body weight of WT and *Ube3a*$^{m-/p+}$ mice fed with a standard diet (sd), as well as *Ube3a*$^{m-/p+}$ mice fed with a LA-enriched diet. Circles represent the average across the mice for each condition ± SEM. **b** Withdrawal responses to pinprick on the hind paw of WT and *Ube3a*$^{m-/p+}$ mice fed with a standard diet (sd), as well as *Ube3a*$^{m-/p+}$ mice fed with a LA-enriched diet (10 trials). Bars are mean ± SEM. One-way ANOVA (F = 0.89; p = 0.42) and Tukey multiple-comparisons test. **c** Response latency to the pressure exerted by an alligator clip placed in the middle of the tail of WT and *Ube3a*$^{m-/p+}$ mice fed with a standard diet (sd), as well as *Ube3a*$^{m-/p+}$ mice fed with a LA-enriched diet. Bars are mean ± SEM. One-way ANOVA (F = 0.011; p = 0.99) and Tukey multiple-comparisons test. **d** Latency of the first nocifensive response (i.e., licking, shaking, biting, guarding, or jumping) to heat (52 ± 0.5 °C) was scored for WT and *Ube3a*$^{m-/p+}$ mice fed with a standard diet (sd), as well as *Ube3a*$^{m-/p+}$ mice fed with a LA-enriched diet. Bars are mean ± SEM. One-way ANOVA (F = 0.42; p = 0.66) and Tukey multiple-comparisons test. **e** Latency of the first nocifensive response to radiant heat (Hargreaves test) applied to the left and right hind paws of WT and *Ube3a*$^{m-/p+}$ mice fed with a standard diet (sd), as well as *Ube3a*$^{m-/p+}$ mice fed with a LA-enriched diet. Bars are mean ± SEM. One-way ANOVA (left: F = 1.16 and p = 0.33; right: F = 0.57 and p = 0.57) and Tukey multiple-comparisons test. **f** Representative blood plasma cytokine profiles and their quantification from WT and *Ube3a*$^{m-/p+}$ mice fed with a standard diet (sd), as well as *Ube3a*$^{m-/p+}$ mice fed with a LA-enriched diet. The array panel includes multiple cytokines: CXCL13/BLC/BCA-1, IL-5, M-CSF, C5a, IL-6, CCL2/JE/MCP-1, G-CSF, IL-7, CCL12/MCP-5, GM-CSF, IL-10, CXCL9/MIG, CCL1/I-309, IL-12 p70, CCL3/MIP-1 α, CCL11/Eotaxin, IL-1, CCL4/MIP-1 β, ICAM-1, IL-16, CXCL2/MIP-2, IFN-γ, IL-17, CCL5/RANTES, IL-1 α/IL-1F1, IL-23, CXCL12/SDF-1, IL-1 β/IL-1F2, IL-27, CCL17/TARC, IL-1ra/IL-1F3, CXCL10/IP-10, TIMP-1, IL-2, CXCL11/I-TAC, TNF-α, IL-3, CXCL1/KC, TREM-1, IL-4. Only those that elicited positive signals are included on the bar graph. Bars are mean ± SD. Kruskal-Wallis (BLC: H = 4.05 and p = 0.13; C5/C5a: H = 6.58 and p = 0.04; sICAM-1: H = 6.39 and p = 0.04; KC: H = 6.24 and p = 0.04; M-CSF: H = 4.01 and p = 0.13; JE: H = 5.42 and p = 0.07; SDF-1: H = 3.48 and p = 0.18; TIMP-1: H = 6.96 and p = 0.03; IFN-γ: H = 4.73 and p = 0.09; IL-16: H = 5.85 and p = 0.054; IL-17: H = 3.85 and p = 0.15; IP-10: H = 3.4 and p = 0.18; RANTES: H = 4.6 and p = 0.1) and Dunn's multiple comparisons test.

Cartoons were created with BioRender.com. n is denoted in each panel. Post hoc p-values are denoted above the bars. Source data are provided as a Source Data file.

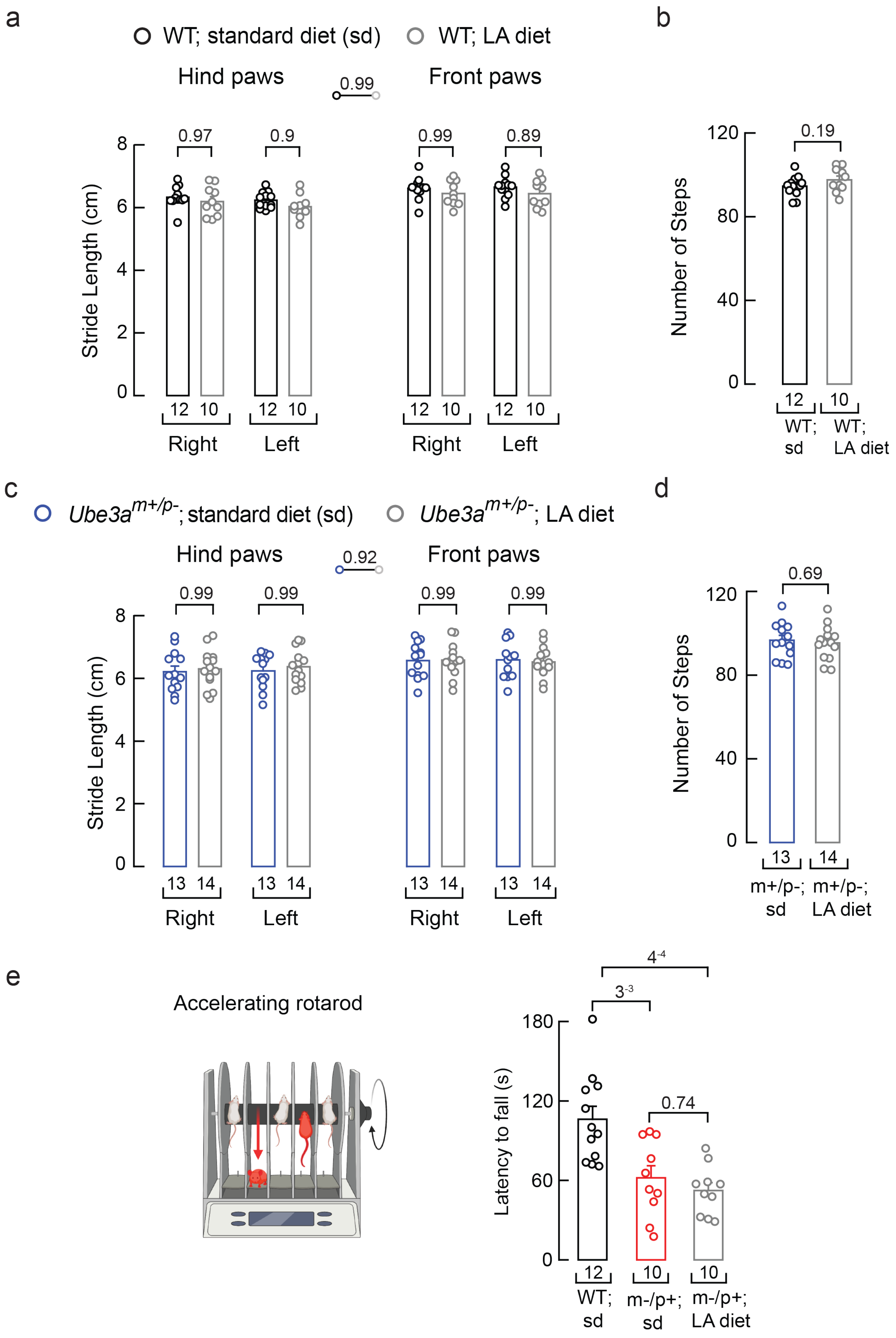
a
WT; standard diet (sd)
WT; LA diet
Hind paws
Front paws
0.99
0.97
0.9
0.99
0.89
Stride Length (cm)
8
6
4
2
0
12 10
12 10
12 10
12 10
Right
Left
Right
Left
b
120
80
40
0
0.19
Number of Steps
12
10
WT; sd
WT; LA diet
c
Ube3a m+/p-; standard diet (sd)
Ube3a m+/p-; LA diet
Hind paws
Front paws
0.92
0.99
0.99
0.99
0.99
Stride Length (cm)
8
6
4
2
0
13 14
13 14
13 14
13 14
Right
Left
Right
Left
d
120
80
40
0
0.69
Number of Steps
13
14
m+/p-; sd
m+/p-; LA diet
e
Accelerating rotarod
4-4
3-3
0.74
180
120
60
0
Latency to fall (s)
12
10
10
WT; sd
m-/p+; sd
m-/p+; LA diet

Supplementary Fig. 8. Gait analyses of the *Ube3a*$^{m+/p-}$ mouse.

a Stride length analysis of hind and front paws (right and left) from WT mice fed with a standard (sd) or LA-enriched diet. Bars are mean ± SEM. Two-way ANOVA (F = 0.049; p = 0.99) and Tukey multiple-comparisons tests. **b** Step number analysis from WT mice fed with a standard (sd) or LA-enriched diet. Bars are mean ± SEM. Two-tailed unpaired t-test (t = 1.34). **c** Stride length analyses of hind and front paws (right and left) from *Ube3a*$^{m+/p-}$ mice fed with a standard (sd) or LA-enriched diet. Bars are mean ± SEM. Two-way ANOVA (F = 0.17; p = 0.92) and Tukey multiple-comparisons tests. **d** Step number analysis from *Ube3a*$^{m+/p-}$ mice fed with a standard (sd) or LA-enriched diet. Bars are mean ± SEM. Two-tailed unpaired t-test (t = 0.4). **e** Accelerating rotarod performance of WT and *Ube3a*$^{m-/p+}$ mice fed with a standard (sd) or LA-enriched diet. Bars are mean ± SEM. One-way ANOVA (F = 11.58; $p = 2.01^{-4}$) and Tukey multiple-comparisons tests. The left cartoon was created with BioRender.com. n is denoted in each panel. Post-hoc p-values are denoted in the corresponding panels. Source data are provided as a Source Data file.

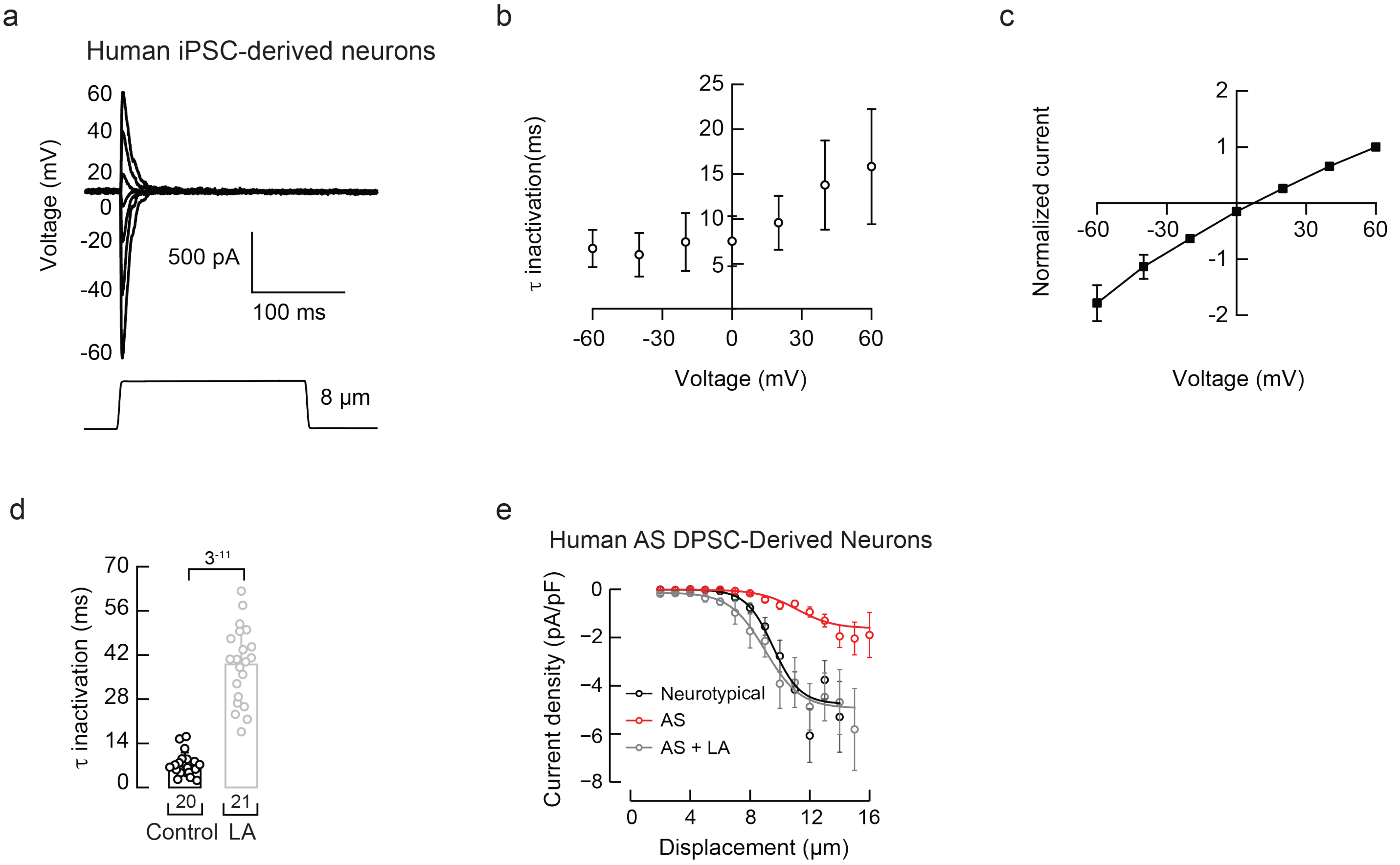

Supplementary Fig. 9. PIEZO2 mediates mechanically evoked currents in human iPSC-derived sensory neurons, and LA increases mechanocurrents in DPSC-derived neurons from AS individuals.

a Representative whole-cell patch-clamp recordings (with voltage pulses ranging from -60 to +60 mV) of human iPSC-derived sensory neurons elicited by mechanical stimulation (bottom). **b** Time constants of inactivation elicited by maximum displacement with voltage pulses ranging from -60 to +60 mV. Circles are mean ± SD. n = 3. **c** Normalized current-voltage relationship of human iPSC-derived sensory neuron mechano-dependent currents, as determined by whole-cell patch-clamp experiments. Reversal potential (+ 7.75 mV). Squares are mean ± SD. n = 3. **d** Time constants of inactivation elicited by maximum displacement of control and LA-treated iPSC-derived sensory neurons. Bars are mean ± SD. Two-tailed unpaired t-test with Welch's correction

(t = 11.48). n is denoted in the panel. p-value is denoted above the bars **e** Current density responses to the displacement of human neurotypical and AS DPSC-derived neurons, with or without LA supplementation. A Boltzmann function was fitted to the data. Each circle represents the mean ± SEM. For neurotypical, n = 21, 21, 21, 21, 21, 21, 21, 21, 21, 19, 14, 8, 6, 3, per displacement. For AS, n = 31, 31, 31, 31, 31, 31, 31, 31, 29, 24, 21, 17, 12, 8, 5, per displacement. For AS + LA, n = 21, 21, 21, 21, 21, 21, 21, 19, 19, 16, 13, 7, 5, 4, per displacement. Source data are provided as a Source Data file.
