# Supplementary material for: Linoleic acid improves PIEZO2 dysfunction in a mouse model of Angelman Syndrome
Source: Nat Commun. 2023 Mar 1;14:1167. doi: 10.1038/s41467-023-36818-0 (PMC9977963; doi:10.1038/s41467-023-36818-0)
Supplement: Supplementary file 3 — Reporting Summary [file 41467_2023_36818_MOESM3_ESM.pdf]

nature portfolio

Corresponding author(s): Julio F. Cordero-Morales, Ph.D.
Valeria Vásquez, Ph.D.

Last updated by author(s): Jan 18, 2023

Reporting Summary

Nature Portfolio wishes to improve the reproducibility of the work that we publish. This form provides structure for consistency and transparency in reporting. For further information on Nature Portfolio policies, see our Editorial Policies and the Editorial Policy Checklist.

Statistics

For all statistical analyses, confirm that the following items are present in the figure legend, table legend, main text, or Methods section.

n/a	Confirmed	
☐	☒	The exact sample size (n) for each experimental group/condition, given as a discrete number and unit of measurement
☐	☒	A statement on whether measurements were taken from distinct samples or whether the same sample was measured repeatedly
☐	☒	The statistical test(s) used AND whether they are one- or two-sided<br>*Only common tests should be described solely by name; describe more complex techniques in the Methods section.*
☐	☒	A description of all covariates tested
☐	☒	A description of any assumptions or corrections, such as tests of normality and adjustment for multiple comparisons
☐	☒	A full description of the statistical parameters including central tendency (e.g. means) or other basic estimates (e.g. regression coefficient) AND variation (e.g. standard deviation) or associated estimates of uncertainty (e.g. confidence intervals)
☐	☒	For null hypothesis testing, the test statistic (e.g. F, t, r) with confidence intervals, effect sizes, degrees of freedom and P value noted<br>*Give P values as exact values whenever suitable.*
☒	☐	For Bayesian analysis, information on the choice of priors and Markov chain Monte Carlo settings
☒	☐	For hierarchical and complex designs, identification of the appropriate level for tests and full reporting of outcomes
☒	☐	Estimates of effect sizes (e.g. Cohen's d, Pearson's r), indicating how they were calculated

Our web collection on statistics for biologists contains articles on many of the points above.

Software and code

Policy information about availability of computer code

Data collection	For electrophysiology: Clampex v10.4.2.0 (Molecular Devices, LLC). For qPCR data: Bio-Rad CFX Maestro 1.0 (Bio-Rad). For protein expression determination (westerns): ChemiDoc Touch Imaging System (Bio-Rad). For confocal micrographs: ZEN2010 software (Zeiss). For differential scanning calorimetry (thermographs): NanoDSC v4.8.2 (TA Instruments). For gait dynamics: CatWalkXT v:10.0.408 (Noldus, USA). For Pinprick: Data collected manually by the investigator. For Tail-Clip: Data collected manually by the investigator and time measured with a chronometer. For Hot Plate: Harvard Apparatus. For Hargreaves: Paw Thermal Stimulator (UC, San Diego). For Rotorod: Rotor-rod (SD Instruments).
Data analysis	For electrophysiology: Clampfit v10.4.2.0 (Molecular Devices, LLC). For plotting: OriginPro 2018 (64-bit) v:b9.5.1.195 (OriginLab Coorporation) and DataGraph v. 5.0. For statistical analyses: GraphPad Instat v.3.10 (GraphPad Software, Inc), OriginPro 2018 (64-bit) v:b9.5.1.195 (OriginLab Coorporation), and EstimationStats (Open software and code available in: estimationstats.com; property of Adam Claridge-Chang and Joses Ho). For qPCR data: Bio-Rad CFX Maestro 1.0 (Bio-Rad). For protein expression determination (westerns): Image Lab Software v6.1.0 (Bio-Rad). For confocal micrographs: Fiji Image J v.2.3.0/1.53q. For differential scanning calorimetry (thermographs): Nano Analyze v3.12.0 (TA Instruments). For gait dynamics: CatWalkXT v:10.0.408 (Noldus, USA) and OriginPro (OriginLab). For Pinprick: Excel (v:2211; Microsoft Corp.) and OriginPro (OriginLab). For Tail-Clip: Excel and OriginPro (OriginLab). For Hot Plate: Excel and OriginPro (OriginLab). For Hargreaves: Excel and OriginPro (OriginLab). For Rotarod: Rotor-rod (SD Instruments) and OriginPro (OriginLab).

For manuscripts utilizing custom algorithms or software that are central to the research but not yet described in published literature, software must be made available to editors and reviewers. We strongly encourage code deposition in a community repository (e.g. GitHub). See the Nature Portfolio guidelines for submitting code & software for further information.

Data

Policy information about availability of data

All manuscripts must include a data availability statement. This statement should provide the following information, where applicable:

- Accession codes, unique identifiers, or web links for publicly available datasets
- A description of any restrictions on data availability
- For clinical datasets or third party data, please ensure that the statement adheres to our policy

Source data are provided with this paper. The source data underlying Figures and Supplementary Figures are provided as a Source Data file available at Figshare. DOI: 10.6084/m9.figshare.20038184

Human research participants

Policy information about studies involving human research participants and Sex and Gender in Research.

Reporting on sex and gender	The differentiation of the de-identified DPSC lines into neurons for molecular studies is considered non-human subjects research under exemption #7 (II.111(a)(8). The PHI has been stripped from the biospecimens in the repository for secondary research studies, and each sample has been de-identified using a sample identifier.
Population characteristics	N/A.
Recruitment	N/A
Ethics oversight	Protocols for human iPSC-derived neurons were reviewed and approved by approved by the IRB at the National Institute of Neurological Disorders and Stroke Intramural Research Program.

Note that full information on the approval of the study protocol must also be provided in the manuscript.

Field-specific reporting

Please select the one below that is the best fit for your research. If you are not sure, read the appropriate sections before making your selection.

☒ Life sciences ☐ Behavioural & social sciences ☐ Ecological, evolutionary & environmental sciences

For a reference copy of the document with all sections, see nature.com/documents/nr-reporting-summary-flat.pdf

Life sciences study design

All studies must disclose on these points even when the disclosure is negative.

Sample size	No sample-size calculation was performed. We obtained data for more than 4 samples per condition (at least three different days). The sample sizes were deemed sufficient based on studies that used similar methodologies on the cell biology and physiology fields (Ma et al, 2023; Ma et al, 2018).<br><br>- Ma S, Cahalan S, LaMonte G, Grubaugh ND, Zeng W, Murthy SE, Paytas E, Gamini R, Lukacs V, Whitwam T, Loud M, Lohia R, Berry L, Khan SM, Janse CJ, Bandell M, Schmedt C, Wengelnik K, Su AI, Honore E, Winzeler EA, Andersen KG, Patapoutian A. Common PIEZO1 Allele in African Populations Causes RBC Dehydration and Attenuates Plasmodium Infection. Cell. 2018 Apr 5;173(2):443-455.e12. doi: 10.1016/j.cell.2018.02.047. Epub 2018 Mar 22. PMID: 29576450; PMCID: PMC5889333.<br><br>- Ma S, Dubin AE, Romero LO, Loud M, Salazar A, Chu S, Klier N, Masri S, Zhang Y, Wang Y, Chesler AT, Wilkinson KA, Vásquez V, Marshall KL, Patapoutian A. Excessive mechanotransduction in sensory neurons causes joint contractures. Science. 2023 Jan 13;379(6628):201-206. doi: 10.1126/science.add3598. Epub 2023 Jan 12. PMID: 36634173.
Data exclusions	For electrophysiological experiments we excluded from the analyses (exclusion criteria were pre-established): Recordings with leak currents > 200pA, with access resistance >10MΩ, and cells with giga-seals that did not withstand at least five consecutive steps of mechanical stimulation were excluded from analyses. For human DPSC-derived neurons, cells with giga-seals notwithstanding at least four consecutive steps of mechanical stimulation were excluded. For CatWalk experiments (exclusion criteria were pre-established): runs shorter than 0.5 seconds, longer than 5 seconds, and with a speed variation higher than 60% across the run were excluded. For Tail-Clip: A cut-off latency of 60 s was imposed to avoid tissue damage. Animals that did not respond at this time were excluded. For Rotarod: Mice that fell 3 times before 10 seconds or performing 2 full passive rotations clinging are excluded.
Replication	All attempts at replication were successful. Experiments were performed at least 3 times in different days with different/independent preparations.
Randomization	The experiments were not randomized. For electrophysiological experiments, daily measurements included the control and treated samples, and only cells with good surface attachment were chosen (e.g., healthy morphology and visible pseudopodia). Transfected cells had a

fluorescent transfection marker, and cells that endogenously express PIEZO2 (neurons and MCC13 cells) were chosen indiscriminately. Cell size was taken into account by normalizing each cell's current magnitude (pA) by their capacitance (pF; which is proportional to the cell size). For behavioral experiments, we measured the behavior of all individuals corresponding to their genotype and treatment. Mice were tested in no particular order.

Blinding

For electrophysiology, the investigator was blind to genotype and treatment. For behavioral assays, the investigator was blind when possible; however, diet smell and consistency can be easily identified. Moreover, the fur of animals on oily-based diets (i.e., linoleic acid) looks shinier than when fed with other diets.

Reporting for specific materials, systems and methods

We require information from authors about some types of materials, experimental systems and methods used in many studies. Here, indicate whether each material, system or method listed is relevant to your study. If you are not sure if a list item applies to your research, read the appropriate section before selecting a response.

Materials & experimental systems

n/a	Involved in the study
☐	☒ Antibodies
☐	☒ Eukaryotic cell lines
☒	☐ Palaeontology and archaeology
☐	☒ Animals and other organisms
☒	☐ Clinical data
☒	☐ Dual use research of concern

Methods

n/a	Involved in the study
☒	☐ ChIP-seq
☒	☐ Flow cytometry
☒	☐ MRI-based neuroimaging

Antibodies

Antibodies used

Mouse monoclonal anti-UBE3A (1:1,000; Sigma-Aldrich Cat# SAB1404508, RRID:AB_10740376), mouse monoclonal anti-actin for MCC13 cells (1:5,000; Bio-Rad Cat# MCA358GT, RRID:AB_323521), mouse monoclonal anti-actin for DRGs (1:1,000; Cytoskeleton Cat# AAN02, RRID:AB_2884962), goat polyclonal anti-mouse IgG H&L (HRP) (1:20,000; Abcam Cat# ab205719, RRID:AB_2755049), rabbit polyclonal anti-human PIEZO2 (1:1,000; abcepta Cat# AP16313b, RRID:AB_11136435), rabbit polyclonal anti-cofilin (1:1,000; abcepta Cat# AP53892, RRID:AB_2923306), rabbit monoclonal anti-cofilin for DRGs (1:1,000; Cell Signaling Technology Cat# 5175, RRID:AB_10622000), and goat anti-rabbit IgG HL-HRP conjugated (1:10,000; Bio-Rad Cat# 1706515, RRID:AB_2617112) antibodies were used for western blots. For cofilin ubiquitination assay, the following primary antibodies were used: mouse monoclonal anti-GFP antibody (1:1,000; Roche Cat# 11814460001, RRID:AB_390913), mouse monoclonal anti-FLAG M2-HRP conjugated antibody (1:1,1000; Sigma-Aldrich Cat# A8592, RRID:AB_439702), and mouse monoclonal anti-UBE3A (1:1,1000; clone E6AP-300) antibody (1:1,1000; Sigma-Aldrich Cat# E8655, RRID:AB_261956). The following secondary antibodies were used: goat anti-mouse-HRP-labeled antibody (1:4,000; Thermo Fisher Scientific Cat# 62-6520, RRID:AB_2533947) and goat anti-mouse IRDye-800CW (1:8,000; LI-COR Biosciences Cat# 926-32210, RRID:AB_621842).

Validation	Primary antibodies used for western blot:<br>1- Mouse monoclonal anti-UBE3A (1:1,000; Sigma-Aldrich Cat# SAB1404508, RRID:AB_10740376). According to the RRID Portal, this antibody has been used in PMID:30364390 and PMID:27339004. Results in Supplementary Figures 2a, 2c-d validate this antibody.<br>2- Mouse monoclonal anti-actin for MCC13 cells (1:5,000; Bio-Rad Cat# MCA358GT, RRID:AB_323521). Results in Supplementary Figures 3b-c validate this antibody.<br>3- Mouse monoclonal anti-actin for DRGs (1:1,000; Cytoskeleton Cat# AAN02, RRID:AB_2884962). According to the manufacturer's website, this antibody has been used in PMID: 35440627, PMID: 35358093, PMID: 33940656, PMID: 33896194, PMID: 34796874.<br>4- Rabbit polyclonal anti-human PIEZO2 (1:1,000; abcepta Cat# AP16313b, RRID:AB_11136435). According to the manufacturer's website, this antibody was validated by western blot analysis of PIEZO2 expression in HepG2 and A549 whole cell lysates, and their antibodies undergo a rigorous validation process by Abcepta scientists. Results in Figure 2b and Supplementary Figure 2b, 2e validate this antibody.<br>5- Rabbit polyclonal anti-cofilin (1:1,000; abcepta Cat# AP53892, RRID:AB_2923306). According to the manufacturer's website, this antibody was validated by western blot analysis of cofilin expression in K562, SHSY5Y, and rat muscle whole cell lysates, and their antibodies undergo a rigorous validation process by Abcepta scientists.<br>6- Rabbit monoclonal anti-cofilin for DRGs (1:1,000; Cell Signaling Technology Cat# 5175, RRID:AB_10622000). According to the RRID Portal, this antibody has been used in PMID:34038737, PMID:34148098, PMID:33513358, PMID:33979620, PMID:33856648, PMID:33657382, PMID:34107066, PMID:32553168, PMID:33400922, PMID:32521269, PMID:32934075, PMID:31668800, PMID:32139584, PMID:32870157, PMID:31679934, PMID:31340145, PMID:29246925, PMID:27216191. According to the manufacturer's website, this antibody is highly specific and rigorously validated in-house.<br>7- Mouse monoclonal anti-GFP antibody (1:1,000; Roche Cat# 11814460001, RRID:AB_390913). According to the RRID Portal, this antibody has been used in 283 publications, the most recent ones being PMID:35231447, PMID:35240051, and PMID:35085497. According to the manufacturer's website, this antibody is tested for functionality and purity relative to a reference standard to confirm the quality of each new reagent preparation.<br>8- Mouse monoclonal anti-FLAG M2-HRP conjugated antibody (1:1,000; Sigma-Aldrich Cat# A8592, RRID:AB_439702). According to the RRID Portal, this antibody has been used in 200 publications, the most recent ones being PMID:35015570, PMID:35314674, PMID:35310945.<br>9- Mouse monoclonal anti-UBE3A (clone E6AP-300) antibody (1:1,000; Sigma-Aldrich Cat# E8655, RRID:AB_261956). According to the RRID Portal, this antibody has been used in PMID:3210937, PMID:30020076, PMID:29979964, and PMID:29354033. Results in Supplementary figure 3f validate this antibody.

Eukaryotic cell lines

Policy information about cell lines and Sex and Gender in Research

Cell line source(s)	Neuro-2a (N2A, ATCC CCL-131) from ATCC; Piezo1 knock-out mouse N2A cells were a gift from Dr. Gary Lewin (produced from N2A, ATCC CCL-131 from ATCC; Reference: Moroni, M., Servin-Vences, M.R., Fleischer, R. et al. Voltage gating of mechanosensitive PIEZO channels. Nat Commun 9, 1096 (2018). https://doi.org/10.1038/s41467-018-03502-7); MCC13 cells were purchased from Sigma Aldrich (10092302-1VL); and HEK293T (293T; ATCC CRL-3216).<br><br>Dental pulp stem cells (DPSC): The differentiation of the de-identified DPSC lines into neurons for molecular studies is considered non-human subjects research under exemption #7 (II.111(a)(8). The PHI has been stripped from the biospecimens in the repository for secondary research studies, and each sample has been de-identified using a sample identifier. The collection of these DPSC lines for our repository is IRB approved under protocol 10-00878-XP.
Authentication	DPSC-derived neurons from individuals with Angelman syndrome display both molecular and cellular features characteristic of Angelman syndrome, as previously reported (Goorha and Reiter, 2017, Curr Protoc Hum; and Chen et al., 2020, JCI Insight; Urraca et al., 2018, Mol Autism), MCC13,<br><br>Cell lines that were not authenticated in the lab: Mouse neuro-2a (N2A; catalog number CCL-131; American Type Culture Collection; ATCC®), Piezo1 knock-out mouse N2A (Piezo1–/– N2A) (from Dr. Gary R. Lewin, Moroni et al 2018), and MCC13 cells (Cell Bank Australia reference number: CBA1338; from Sigma)<br><br>Moroni M, Servin-Vences MR, Fleischer R, Sánchez-Carranza O, Lewin GR. Voltage gating of mechanosensitive PIEZO channels. Nat Commun. 2018 Mar 15;9(1):1096. doi: 10.1038/s41467-018-03502-7. PMID: 29545531; PMCID: PMC5854696.
Mycoplasma contamination	Cells were not tested for mycoplasma contamination.
Commonly misidentified lines<br>(See ICLAC register)	No commonly misidentified cell lines were used on this study.

Animals and other research organisms

Policy information about studies involving animals; ARRIVE guidelines recommended for reporting animal research, and Sex and Gender in Research

Laboratory animals	Mice/Strains: C57BL/6 (Stock No. 000664); C57BL/6 Ubeatm1Alb (B6 AS; Stock No. 016590) for maternal and paternal transmission.<br>Age of animals use or experimentation was from 6 weeks to 5 months.<br>Mice were housed with a 12 h light/dark cycle at 21°C with 40-60% humidity, with food and water ad libitum

Wild animals	No wild animals were used in the study.
Reporting on sex	Mice sex was reported on the manuscript. Female and male animals were used for electrophysiological experiments, and sex-based analyses are indicated on the manuscript and in the source data file. For behavioral analysis, only male mice were used and as such reported on the methodology.
Field-collected samples	No field collected samples were used in the study.
Ethics oversight	Protocols for mice have been reviewed and approved by the IACUC. The University of Tennessee maintains an AALAC-accredited facility that is staffed by 3 full time veterinarians, 2 of whom are board-certified in laboratory animal care.

Note that full information on the approval of the study protocol must also be provided in the manuscript.
